# Tribute to Dr. J. M. Johnson

**Published:** 1886-06-20

**Authors:** 


### TRIBUTE TO DR. J. M. JOHNSON.

In pursuance to a call, a number of physicians met at the hall of the Atlanta Society of Medicine May, 19th, expressive of their respect to the memory of the late Dr. J. M. Johnson.

Dr J. F. Alexander was called to the chair and Dr. Arch Avary requested to act as Secretary.

On motion, a committee was appointed to draft resolutions and the following submitted and adopted:

Whereas, By the providence of God death has removed from our midst our distinguished brother, Dr. John M. Johnson, for so many years a leader in his profession, and by his wide knowledge a guide to his brethren and a benefactor to humanity; therefore be it,

Resolved, That we, members of the medical profession of Atlanta, assembled to do honor to his memory, do recognize in his death that a great and good man has gone to his just reward, and that in his long and blameless life we find an example worthy of our emulation and reverence.

Resolved, That we extend to his family and relatives our warm sympathy in this their great bereavement, believing at the same time that their sorrow must be tempered by the realization of the fact that his well rounded life reached its rich fruition, and left behind many hearts to mourn his loss and remember his kindly and charitable ministrations.

Resolved, That the medical profession, as a mark of respect, attend his funeral in a body.

Resolved, That these resolutions be published in the daily papers, and in the Southern Medical Record and Atlanta Medical Journal.

WM. PERRIN NICHOLSON, M.D.,
E. L. CONNOLLY, M.D.,
J. G. EARNEST, M.D., } Committee.
J. C. AVARY, M.D.,
N. O. HARRIS, M.D.,

In the month of May, 1886, at Atlanta, Ga., there went home to his eternal rest one of the most popular and distinguished physicians of our city—Dr. JOHN M. JOHNSON, in the seventy fifth year of his age.

To appreciate a man like Dr. Johnson one must know him intimately and feel the full influence of his marked coaracter. The firmness of his principles, the import of his purposes, the generosity of his disposition, and his cheerful, hopeful views of life, made a combination of characteristic forces in a man whose influence was great, and to be remembered both socially and professionally. He was also one of those rare members of our profession who, while keeping thoroughly informed upon the medical progress and discoveries of the age, found time to explore fields of philosophical, scientific and scholastic literature. He was an appreciative lover of fine literary productions, and was himself an accomplished scholar and entertaining conversationalist.

Dr. Johnson was born January 15, 1812, in Livingston County, Ky. His father Dr. James Johnson, a man of forcible character and great will-power, was prominently identified with the early history of that State. The family is of good English blood, and is descended from Sir James Johnson, knighted in 1671 by Charles II. The immediate ancestor of the American branch of the family was a Thomas Johnson, who came over to Maryland in 1700. Roger Johnson, one of his grandsons, was the grandfather of Dr. Johnson, the subject of this sketch. Joshua Johnson, another grandson, was the father of Mrs. John Quincy Adams, and the grandfather of Charles Francis Adams. Still another grandson, Thomas Johnson, was Governor of Maryland from 1775 to 1779.

In 1827 Dr. John M. Johnson began the study of medicine under Dr. Miller, of Madisonville, Ky., who quickly discovered that his young student had great talent for the profession he had chosen. The eminent preceptor gave unusual care and attention to the subject of his instructions, and predicted for him the great success that crowned him in the mature advancement of later years.

At some period between the years 1840–'45 Dr. Johnson won the high appreciation and gratitude of his friends, patrons, and professional brethren by the skillful and successful treatment of an epidemic known as Milk-sickness. His ably written articles upon the disease and its causes were copied extensively in this country, and by the London *Lancet*, with favorable comment. Always genial and popular, he was several times urged by his friends for Governor of Kentucky, but always declined, as he preferred the duties of his profession ; but he filled several important positions of public trust, and was chosen to carry the electoral vote of Kentucky to Washington City when General Taylor was made President of the United States. In the stirring political events that ushered in the civil war, he took a prominent part in the Southern ranks, and the State Rights party. He warmly advocated the Secession Ordinance, and was a member of the State Senate when the war began in 1861. His zeal caused the Federal authorities to order his arrest, but by a fortunate chance he escaped capture, and joined the division of the Southern forces under Gen. Albert Sidney Johnston. From that time he was a medical officer in the Confederate Army until the close of the war. He then located in Atlanta, where he lived until the day of his death.

Dr. Johnson was first married, in 1832, to Miss Elizabeth Earle, daughter of Col. John Baylis Earle, of Earle's Ferry, S. C. Ten children were the fruit of this union, of whom only three are living—Mrs. Gen. G. B. Cosby, of Sacramento City, Cal. ; Mrs. Col. E. R. Wire, of Greenville, Ky., and Mrs. J. L.

Byers, of Atlanta, Ga. In 1864 Doctor Johnson contracted a second marriage with Mrs. Mary W. Erwine, a sister of the two distinguished and lamented brothers, Gen. Howell Cobb, and Gen. Thos. Cobb, of Georgia. She was a noble and accomplished woman, of high social position and much noted for her patriotic zeal and the generous, warmhearted usefulness in benevolent movements and all noble enterprizes. P.

